# External validation of the CRASS score for predicting good neurological outcome in out-of-hospital cardiac arrest: analysis from cardiac-origin and non-cardiac origin cohorts

**DOI:** 10.1186/s12873-026-01472-4

**Published:** 2026-01-16

**Authors:** Chih-Wei Sung, Ching-Yu Chen, Cheng-Yi Fan, Yi-Chien Kuo, Chun-Hsiang Huang, Sih-Shiang Huang, Chi-Hsin Chen, Chien-Tai Huang, Yi-Ju Ho, Chun-Ju Lien, Wei-Tien Chang, Edward Pei-Chuan Huang

**Affiliations:** 1 Department of Emergency Medicine, National Taiwan University Hospital Hsin-Chu Branch, Hsinchu, Taiwan; 2 Department of Emergency Medicine, College of Medicine, National Taiwan University, Taipei, Taiwan; 3 Department of Emergency Medicine, National Taiwan University Hospital Yun-Lin Branch, Yunlin, Taiwan; 4 Institute of Molecular Medicine, National Tsing Hua University, Hsinchu, Taiwan; 5 Section of Emergency Medicine, Department of Medicine, National Taiwan University Cancer Center, Taipei, Taiwan; 6 Graduate Institute of Clinical Medicine, College of Medicine, National Taiwan University, Taipei, Taiwan; 7 Graduate Institute of Biomedical Informatics, College of Medical Science and Technology, Taipei Medical University, Taipei, Taiwan; 8 Institute of Epidemiology and Preventive Medicine, College of Public Health, National Taiwan University, Taipei, Taiwan; 9 Department of Emergency Medicine, National Taiwan University Hospital, Taipei, Taiwan

**Keywords:** Out-of-hospital cardiac arrest, CRASS score, Neurological outcome, Prediction model, External validation

## Abstract

**Background:**

This study aimed to externally validate the CaRdiac Arrest Survival Score (CRASS) for predicting good neurological outcomes in Asian patients with out-of-hospital cardiac arrest (OHCA), focusing on cardiac-origin and noncardiac-origin cohorts, respectively.

**Methods:**

This multicenter retrospective cohort study, conducted from January 2016 to December 2023 across three hospitals in Taiwan, included patients with OHCA with resuscitation attempts, and excluded those with trauma-related arrests, pediatric cases, or missing data. The CRASS score was calculated for each patient according to the clinical variables at presentation. The outcome involves a good neurological outcome (Cerebral Performance Category (CPC) 1 or 2) at hospital discharge. Predictive performance was evaluated using the area under the receiver operating characteristic curve (AUROC), calibration plots, and other performance metrics, including sensitivity, specificity, and positive and negative predictive values.

**Results:**

This study analyzed 1,311 patients with OHCA (667 cardiac-origin and 644 noncardiac-origin). The AUROC for predicting good neurological outcomes was 0.770 (95% confidence interval [CI]: 0.733–0.807) in the cardiac-origin cohort compared with 0.729 (95% CI: 0.661–0.796) in the noncardiac-origin cohort. The CRASS score exhibited better predictive performance in patients with cardiac origin, with an optimal cut-off value of 1.45, thereby supporting more aggressive treatment. The score in patients with noncardiac origin was more effective in predicting poor neurological outcomes, with an optimal cut-off value of − 1.47, favoring life support withdrawal.

**Conclusions:**

The CRASS score is effective for predicting good neurological outcomes in patients with cardiac-origin OHCA but is more suited to guiding treatment withdrawal in noncardiac-origin cases.

**Clinical trial number:**

Not applicable.

**Supplementary Information:**

The online version contains supplementary material available at 10.1186/s12873-026-01472-4.

## Introduction

Neuroprognostication in patients with out-of-hospital cardiac arrest (OHCA) is crucial for guiding care decisions [1, 2], managing family expectations, and ensuring ethical choices. Accurate prognostication helps tailor treatment plans, from aggressive interventions to palliative care, and prevents premature life-sustaining therapy withdrawal in patients with potential for recovery [3]. Additionally, it plays a crucial role in optimizing resource allocation in intensive care units and supports families in making informed decisions. Moreover, neuroprognostication improves research by refining predictive models and advancing the knowledge of neurological outcomes after cardiac arrest, thereby ultimately enhancing survival and recovery rates [4–6]. Hence, several scores have been established for predicting neurological outcomes in patients with OHCA [7–11].

The CaRdiac Arrest Survival Score (CRASS), developed by the German Resuscitation Registry [12], was established to predict good neurological outcomes post-OHCA according to data from the registry [13]. A major limitation of the original CRASS score was its registry-based nature, which causes potential dropouts or missing variables. Liu et al. (2022) performed an external validation of the CRASS score for good neurological outcomes in a Singapore cohort [14]. However, issues, such as differing variable definitions, varying data structures, and certain variable removal made this seem more like a modified CRASS score version, rather than a direct validation in a different population. Additionally, the etiology of cardiac arrest significantly differed between previous studies. A comprehensive external validation of the CRASS score system, strictly adhering to the original variable definitions, remains not conducted in non-European populations.

Validation in a non-European population is essential because the CRASS score was originally derived from the German Resuscitation Registry, reflecting Western demographic and prehospital characteristics. Differences in OHCA etiology, emergency medical service systems, and in-hospital postarrest care between Europe and Asia may affect the model’s calibration and clinical applicability. This study aimed to externally validate the CRASS score in an Asian (Taiwanese) population to determine whether it maintains predictive accuracy across diverse etiologies and healthcare systems. Clinically, this validation seeks to clarify whether CRASS can help identify (1) cardiac-origin OHCA patients who could benefit from aggressive management and (2) noncardiac-origin patients for whom early prognostic information may guide communication about treatment goals.

## Methods

### Study design and setting

This multicenter retrospective cohort study, conducted from January 2016 to December 2023 at the National Taiwan University Hospital and its two affiliated hospitals, the Hsinchu and Yunlin branches, obtained data from the National Taiwan University Hospital’s Out-of-Hospital Cardiac Arrest Research Database (NTUH-HYCARD). Data included detailed comprehensive records from the main and the Hsinchu and Yunlin branches specifically customized for OHCA research, ensuring a strong foundation for data analysis. The study was conducted in accordance with the tenets of the 2013 revision of the Declaration of Helsinki.

### Inclusion and exclusion criteria

Eligible patients were adults with emergency medical services (EMS)-attended, non-traumatic out-of-hospital cardiac arrest who received resuscitation attempts and subsequently achieved ROSC (either prehospital or in the emergency department) and were admitted for post–cardiac arrest care [15]. In Taiwan, EMS personnel are not legally authorized to declare death at the scene or terminate resuscitation upon hospital arrival. Hence, all patients with OHCA managed by EMS are transported to the nearest hospital for continued resuscitation [16]. Also, the EMS system is tiered. Only emergency medical technician–paramedics (EMTPs) are authorized to administer medications in the prehospital setting, whereas some ambulance crews are staffed by providers who deliver basic life support only; therefore, prehospital drug administration may vary by crew composition.

The exclusion criteria were (1) patients whose cardiac arrest was trauma-related, (2) those aged < 18 years, and (3) patients with missing outcome information.

### The CRASS score

The CRASS score, developed by Seewald et al., involves several factors, including age, cardiac rhythm, etiology, chest compression support, adrenaline administration, pre-emergency status (PES), arrest location, amiodarone administration, status at admission, witnessed arrest, cardiopulmonary resuscitation (CPR) duration, and the time between collapse and the start of CPR. It is a multivariable prognostic model developed to estimate the probability of survival with a good neurological outcome after OHCA. The CRASS score is calculated at the patient level using routinely available prehospital and early in-hospital variables collected during resuscitation and on hospital arrival. In the original model, each predictor contributes to a weighted linear predictor, which is then transformed into an individual predicted probability of good neurological outcome using the logistic function [13].

In this study, the CRASS score was applied exactly as defined by Seewald, using the same variables, categories, and logistic regression coefficients. Each variable—including age, initial rhythm, arrest etiology, location, PES, CPR duration, adrenaline and amiodarone use, and time from collapse to CPR—was extracted using the same categorical structure and cut-off points. All original CRASS predictors, including ‘status at admission’ and ‘CPR duration,’ were incorporated as originally defined to ensure methodological fidelity and external comparability.

### Data collection, variables, and definitions

The Research Electronic Data Capture (REDCap) system, which is a secure web-based platform customized for accurate data collection in research [17], was utilized to collect data from both documented and electronic medical records after confirming patient eligibility. Emergency department physicians conducted monthly reviews of the collected information to maintain data quality.

Data variables from the NTUH-HYCARD consisted of demographics, preexisting conditions, and CRASS score indices. The preexisting conditions included hypertension, diabetes mellitus, dyslipidemia, coronary artery disease, congestive heart failure, chronic kidney disease, and chronic obstructive pulmonary disease. These diagnoses were validated through medical records, including consistent medication prescriptions, ongoing treatments, and outpatient follow-up documentation.

The resuscitation events were documented following the definition of the Utstein Resuscitation Registry Template for the CRASS variables [18]. However, the articles by Seewald [13] and Liu [14] did not explicitly define the PES category; thus, we initially included 22 common diseases in Taiwan for analysis in this study (Supplementary Table 1) [19]. Our validation used the same variable definitions and weighting without re-estimating coefficients. The univariate regression described in this study was used only to categorize 22 locally prevalent comorbidities into ‘major’ or ‘minor’ disease groups under the pre-emergency status (PES) variable, as the original publication did not specify this classification in detail. This exploratory step ensured compatibility with the CRASS framework and did not modify the variable set or model structure. Specifically, we categorized diseases with a *p*-value of < 0.05 (including diabetes, coronary artery disease, hyperlipidemia, cerebrovascular disease, psychiatric disorders, and cancer) as “major diseases,” whereas the remaining conditions were classified as “minor diseases.” Patients with one or more major diseases were classified as not belonging to the “without prior disease (+ 0.5)” or “with minor disease (+ 0.2)” categories. Collapse-to-CPR interval is defined as the time from collapse (or recognition of arrest) to the initiation of CPR. In addition, CPR duration is defined as the time from the initiation of CPR to sustained ROSC.

### Outcomes

The primary outcome of the study includes a good neurological outcome, assessed using the Glasgow–Pittsburgh Cerebral Performance Category (CPC) scores during hospital discharge after postarrest care [20]. CPC scores of 1 and 2, indicating good cerebral performance and recovery, were categorized as good neurological outcomes.

### Statistical analysis

Patients were classified a priori into cardiac-origin and noncardiac-origin OHCA groups, as previous studies have shown substantial etiological differences in initial rhythm, survival likelihood, and neurological outcome trajectories. The cardiac causes category included acute myocardial infarction, fatal arrhythmia, cardiomyopathy-related arrest, myocarditis, pulmonary embolism, valvular heart diseases, and heart failure complicated with cardiogenic shock. This stratification allowed evaluation of the CRASS score’s performance within clinically distinct populations, recognizing that the original CRASS cohort primarily comprised cardiac-origin cases. The prespecified objective was to assess whether predictive validity differs across these etiologic categories rather than to re-estimate a unified overall model. This classification was identified during monthly meetings led by the principal investigator and senior physicians. They independently reviewed the medical charts, including patient complaints, laboratory findings, and imaging assessments, to establish the final decision. A dedicated statistician, who also validated the data, conducted the statistical analyses with the SAS software version 9.4 (SAS Institute Inc., Cary, NC).

Sample size adequacy for model validation was assessed according to the approach proposed by Riley et al. [21]. Using the observed outcome proportion, c-statistic, and linear predictor parameters of the CRASS model, the minimum required sample size was estimated under three criteria (O/E ratio, calibration slope, and c-statistic precision). The largest required sample size was 1,223, as shown in Supplementary Fig. 1.

The Kolmogorov–Smirnov test was conducted to evaluate the normality of continuous data [22]. Categorical variables were presented as frequencies and percentages and compared using the Chi-square test, whereas Fisher’s exact test was conducted for relative mall sample sizes [23]. Continuous variables were reported as mean ± standard deviation and analyzed with the Student’s *t*-test. Discrimination and calibration analyses were conducted to assess the performance of the CRASS score in this cohort.

Discrimination was assessed by calculating the cross-validated area under the receiver operating characteristic (AUROC) curve, along with its 95% confidence interval (CI). Additionally, the C-statistic and its corresponding 95% CI were identified to further measure the model’s ability to distinguish between outcomes. Calibration was evaluated using calibration plots, comparing the predicted probabilities to the actual event rates across different deciles of risk prediction. DeLong’s test was used to assess the statistical significance of differences between the AUROCs of each model. Sensitivity, specificity, accuracy, positive predictive value (PPV), negative predictive value (NPV), positive likelihood ratio (LR+), and negative likelihood ratio (LR-) were calculated for a comprehensive assessment of the model’s diagnostic performance. A two-tailed statistically significant was set at a *p*-value of < 0.05.

## Results

### Patient enrollment and baseline characteristics

**Fig. 1:**
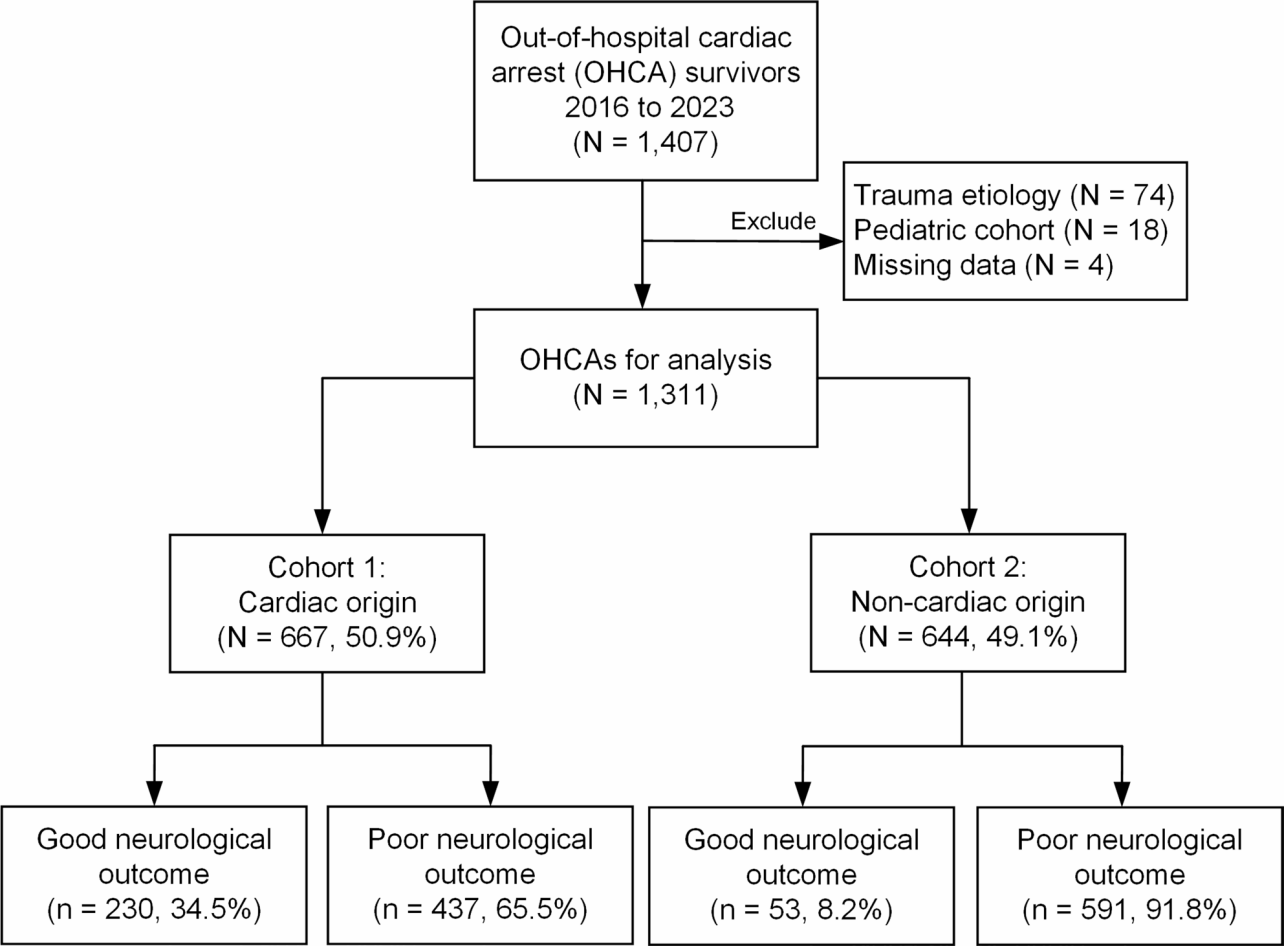
The flowchart of the selection process for OHCA survivors, showing exclusions and classification into cardiac and non-cardiac origin cohorts. The neurological outcome are classified as good or poor in each cohort

Figure 1 illustrates the eligible patient enrollment. The analysis initially considered 1,407 OHCA survivors from 2016 to 2023. The final analysis included 1,311 OHCA cases after excluding cases with trauma etiology (*n* = 74), pediatric patients (*n* = 18), and those with missing data (*n* = 4). These patients were classified into two cohorts: those with a cardiac-origin arrest (cohort 1, *n* = 667, 50.9%) and those with a noncardiac origin (cohort 2, *n* = 644, 49.1%). In cohort 1 (cardiac origin), 34.5% (*n* = 230) achieved a good neurological outcome, whereas 65.5% (*n* = 437) demonstrated a poor neurological outcome. In contrast, only 8.2% (*n* = 53) of cohort 2 (noncardiac origin) achieved a good neurological outcome, whereas 91.8% (*n* = 591) exhibited a poor neurological outcome. Patients with OHCA having a cardiac origin had markedly better neurological outcomes.

Table 1 demonstrates the comparative analysis of patient characteristics between the cardiac-origin and noncardiac-origin cardiac arrest groups. Both cohorts demonstrated that younger patients (≤ 60 years) exhibited a higher rate of good neurological outcomes compared with older patients, with significant differences across age groups in both cohorts. The proportion of males in the group with good neurological outcomes was higher than that in the group with poor neurological outcomes in the cardiac-origin cohort (84.8% vs. 72.5%, *p* < 0.001). No such significant differences were observed in the noncardiac origin group (54.7% vs. 58.7%, *p* = 0.572). Preexisting conditions, such as diabetes mellitus, were more prevalent in the poor outcome group in the cardiac-origin cohort (35.2% vs. 20.0%, *p* < 0.001), but with no significant differences in the noncardiac group (*p* = 0.744). Additionally, patients with good neurological outcomes in both cohorts demonstrated a higher rate of witnessed arrests and initial rhythms of ventricular fibrillation/ventricular tachycardia (Vf/VT). Overall, more variables exhibited significant differences between patients with good and poor neurological outcomes in the cardiac-origin cohort.

**Table 1:** Comparative analysis of patient characteristics and outcomes between cardiac origin and non-cardiac origin cardiac arrest cohorts

	Cohort 1: cardiac origin				Cohort 2: non-cardiac origin			
Variables	Total	Poor outcome	Good outcome	*p*	Total	Poor outcome	Good outcome	*p*
	(*N* = 667)	(*N* = 437)	(*N* = 230)		(*N* = 644)	(*N* = 591)	(*N* = 53)	
Age				< 0.001				0.001
≦ 60 years	294 (44.1)	174 (39.8)	120 (52.2)		187 (29.0)	160 (27.1)	27 (50.9)	
61–70 years	171 (25.6)	107 (24.5)	64 (27.8)		139 (21.6)	125 (21.2)	14 (26.4)	
71–80 years	122 (18.3)	88 (20.1)	34 (14.8)		139 (21.6)	132 (22.3)	7 (13.2)	
81–90 years	63 (9.4)	53 (12.1)	10 (4.3)		144 (22.4)	140 (23.7)	4 (7.5)	
> 91 years	17 (2.5)	15 (3.4)	2 (0.9)		35 (5.4)	34 (5.8)	1 (1.9)	
Sex (Male)	512 (76.8)	317 (72.5)	195 (84.8)	< 0.001	376 (58.4)	347 (58.7)	29 (54.7)	0.572
Pre-existing diseases								
Hypertension	307 (46.0)	208 (47.6)	99 (43.0)	0.262	267 (41.5)	246 (41.6)	21 (39.6)	0.777
Diabetes mellitus	200 (30.0)	154 (35.2)	46 (20.0)	< 0.001	195 (30.3)	180 (30.5)	15 (28.3)	0.744
Coronary artery disease	206 (30.9)	135 (30.9)	71 (30.9)	0.995	83 (12.9)	76 (12.9)	7 (13.2)	0.942
Congestive heart failure	78 (11.7)	50 (11.4)	28 (12.2)	0.780	36 (5.6)	33 (5.6)	3 (5.7)	0.981
Chronic kidney disease	115 (17.2)	85 (19.5)	30 (13.0)	0.037	101 (15.7)	90 (15.2)	11 (20.8)	0.289
Hyperlipidemia	101 (15.1)	56 (12.8)	45 (19.6)	0.021	53 (8.2)	48 (8.1)	5 (9.4)	0.739
COPD	15 (2.2)	12 (2.7)	3 (1.3)	0.233	37 (5.7)	34 (5.8)	3 (5.7)	0.978
Pre-arrest heath condition				0.046				0.953
With relevant disease	382 (57.3)	265 (60.6)	117 (50.9)		388 (60.2)	357 (60.4)	31 (58.5)	
Without prior disease	71 (10.6)	41 (9.4)	30 (13.0)		85 (13.2)	78 (13.2)	7 (13.2)	
With minor disease	214 (32.1)	131 (30.0)	83 (36.1)		171 (26.6)	156 (26.4)	15 (28.3)	
Location of arrest				< 0.001				0.003
Home	279 (41.8)	215 (49.2)	64 (27.8)		360 (55.9)	341 (57.7)	19 (35.8)	
Nursing home	71 (10.6)	44 (10.1)	27 (11.7)		84 (13.0)	70 (11.8)	14 (26.4)	
Working place/sport facility	251 (37.6)	136 (31.1)	115 (50.0)		176 (27.3)	157 (26.6)	19 (35.8)	
Public place	66 (9.9)	42 (9.6)	24 (10.4)		24 (3.7)	23 (3.9)	1 (1.9)	
Witnessed arrest	495 (74.2)	305 (69.8)	190 (82.6)	< 0.001	373 (57.9)	335 (56.7)	38 (71.7)	0.034
Initial cardiac rhythm				< 0.001				< 0.001
Asystole	283 (42.4)	193 (44.2)	90 (39.1)		382 (59.3)	355 (60.1)	27 (50.9)	
PEA	211 (31.6)	160 (36.6)	51 (22.2)		249 (38.7)	226 (38.2)	23 (43.4)	
Vf/VT	173 (25.9)	84 (19.2)	89 (38.7)		13 (2.0)	10 (1.7)	3 (5.7)	
Collapse-to-CPR interval				0.079				0.002
0–1 min	129 (19.3)	75 (17.2)	54 (23.5)		175 (27.2)	150 (25.4)	25 (47.2)	
2–9 min	409 (61.3)	270 (61.8)	139 (60.4)		353 (54.8)	330 (55.8)	23 (43.4)	
≧ 10 min	129 (19.3)	92 (21.1)	37 (16.1)		116 (18.0)	111 (18.8)	5 (9.4)	
CPR duration ≦ 5 min	56 (8.4)	14 (3.2)	42 (18.3)	< 0.001	37 (5.7)	26 (4.4)	11 (20.8)	< 0.001
Mechanical CPR	537 (80.5)	380 (87.0)	157 (68.3)	< 0.001	487 (75.6)	452 (76.5)	35 (66.0)	0.090
Prehospital adrenaline				0.017				0.657
No adrenaline	538 (80.7)	341 (78.0)	197 (85.7)		558 (86.6)	510 (86.3)	48 (90.6)	
< 2 mg	20 (3.0)	10 (2.3)	10 (4.3)		6 (0.9)	5 (0.8)	1 (1.9)	
2–3 mg	75 (11.2)	56 (12.8)	19 (8.3)		51 (7.9)	49 (8.3)	2 (3.8)	
4–5 mg	19 (2.8)	17 (3.9)	2 (0.9)		17 (2.6)	15 (2.5)	2 (3.8)	
6–7 mg	8 (1.2)	7 (1.6)	1 (0.4)		9 (1.4)	9 (1.5)	0 (0.0)	
≧ 8 mg	7 (1.0)	6 (1.4)	1 (0.4)		3 (0.5)	3 (0.5)	0 (0.0)	
Prehospital amiodarone	12 (1.8)	4 (0.9)	8 (3.5)	0.018	0 (0.0)	0 (0.0)	0 (0.0)	
SBP > 90 mmHg at admission	590 (88.5)	374 (85.6)	216 (93.9)	0.001	563 (87.4)	513 (86.8)	50 (94.3)	0.113
CRASS score	0.13 ± 1.40	-0.31 ± 1.30	0.98 ± 1.16	< 0.001	-0.47 ± 1.27	-0.56 ± 1.25	0.50 ± 1.11	< 0.001

Dichotomous and categorical variables were reported as number (percentages), whereas continuous variables were reported as mean ± standard deviation

COPD: chronic obstruction pulmonary disease; CPR: cardiopulmonary resuscitation; CRASS: CaRdiac-Arrest-Survival-Score; PEA: pulseless electrical activity; SBP: systolic blood pressure; Vf: ventricular fibrillation; VT: ventricular tachycardia

The mean CRASS score was 0.13 ± 1.40 in the cardiac-origin cohort. Patients with a good neurological outcome demonstrated significantly higher CRASS scores than those with a poor neurological outcome (0.98 vs. −0.31, *p* < 0.001). Similarly, the mean CRASS score was − 0.47 ± 1.27 in the noncardiac origin cohort, with patients achieving a good neurological outcome demonstrating higher CRASS scores than those with a poor neurological outcome (0.50 vs. −0.56, *p* < 0.001).

### The receiver operating characteristic curve in the cardiac-origin and noncardiac-origin cohorts

**Fig. 2:**
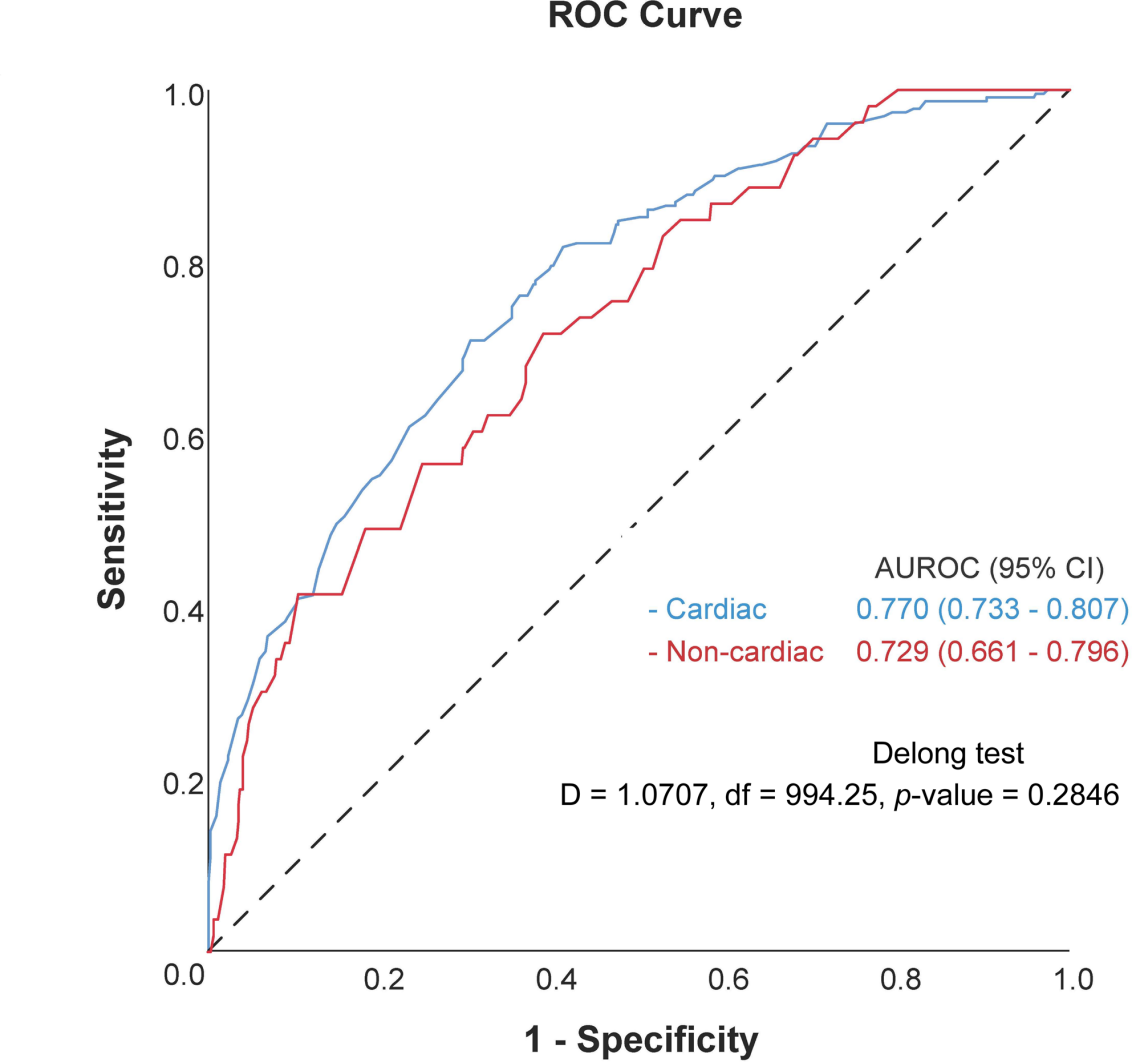
The Receiver Operating Characteristic (ROC) curves in predicting good neurological outcome for cardiac (blue line) and non-cardiac (red line) OHCA patients. The dashed diagonal line represents the line of no discrimination (AUROC = 0.5)

Figure 2 illustrates the receiver operating characteristic (ROC) curve comparing the predictive performance for good neurological outcomes between the cardiac and noncardiac origin OHCA cohorts. The blue line indicates the cardiac-origin cohort, whereas the red line represents the noncardiac-origin cohort. The AUROC values for the cardiac- and noncardiac origin groups were 0.770 (95% CI: 0.733–0.807) and 0.729 (95% CI: 0.661–0.796), which were slightly lower, respectively. These results indicate that the model demonstrates a better predictive performance for neurological outcomes in patients with cardiac-origin OHCA compared with those of noncardiac-origin. Additionally, using DeLong’s test, no statistically significant difference was observed between the AUROC values for the cardiac-origin and noncardiac-origin cohorts (df = 994.25, *p* = 0.285). Supplementary Table 2 presents the advanced association between variables and good neurological outcomes in both cardiac-origin and noncardiac-origin cohorts.

### Trend of performance matrices as a function of the CRASS score

**Fig. 3:**
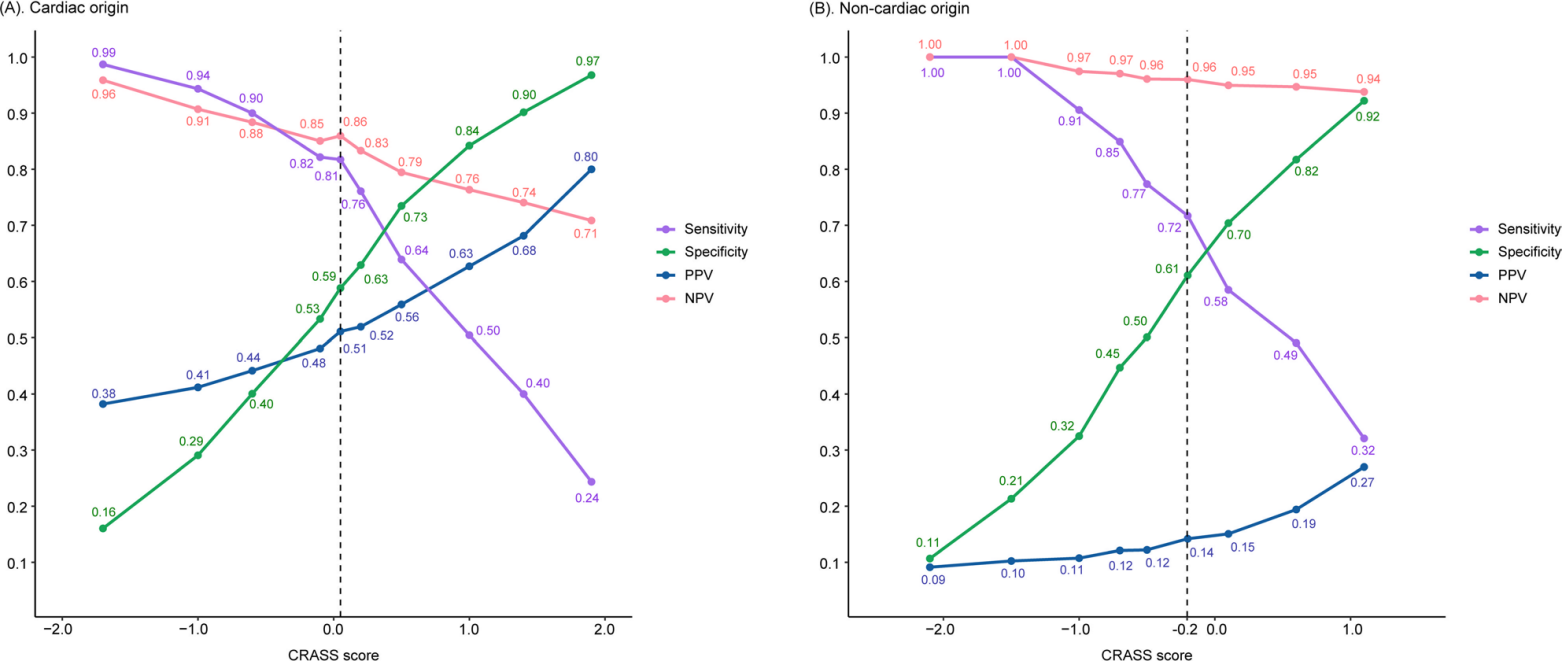
Performance metrics for predicting good neurological outcome across different CRASS score thresholds for (**A**) cardiac-origin and (**B**) non-cardiac origin OHCA patients. The curves represent sensitivity (purple), specificity (green), positive predictive value (PPV, blue), and negative predictive value (NPV, pink) as a function of the CRASS score. The vertical dashed line indicates the optimal threshold for prediction

Figure 3 illustrates the trend between sensitivity, specificity, PPV, and NPV as CRASS score’s functions for both the cardiac- (Panel A) and noncardiac-origin (Panel B) cohorts. Each line was composed of 10 points, with each point representing the 10th, 20th, and up to 90th percentile of the CRASS score for that group, along with an additional point representing the optimal cut-off point. The vertical dashed lines in both panels marked the optimal cut-off points, indicating the score thresholds where the balance between true positives and true negatives was most favorable for each cohort. Because the CRASS score distribution and outcome prevalence differ by etiology, the performance metrics and ‘optimal’ cut-off values are presented for within-cohort interpretation and are not intended for direct comparison across cohorts.

Sensitivity began near 100% at lower CRASS scores and gradually decreased to approximately 60% as the score increased in the cardiac-origin cohort. Conversely, specificity started below 40% and increased steadily, reaching approximately 90% at higher CRASS scores. PPV exhibited a marked increase from 30% to 80%, whereas NPV decreased from 90% to 60% when the CRASS score elevated. The optimal cut-off point for the cardiac-origin cohort was at a CRASS score of approximately 0, where sensitivity and specificity intersect near 80%.

A similar trend was found for the noncardiac-origin cohort, despite slightly different values. The sensitivity decreased from 100% to approximately 40%, whereas the specificity increased from 10% to nearly 90%. The optimal cut-off point for the noncardiac cohort was determined at a CRASS score of approximately 0.6, where sensitivity and specificity balance at approximately 70%.

### Calibration plot for the agreement assessment

**Fig. 4:**
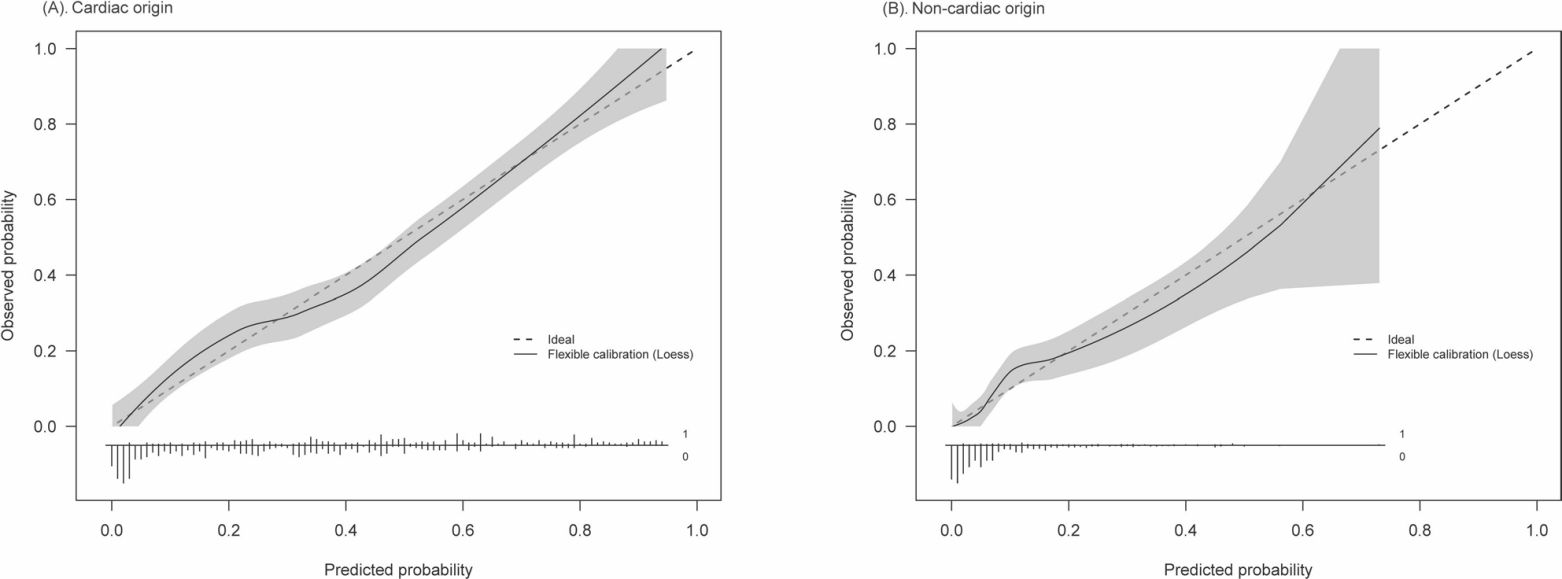
Calibration plots for the predicted versus observed probabilities of good neurological outcome in OHCA patients with (**A**) cardiac-origin and (**B**) non-cardiac origin. The dashed line indicates the ideal calibration, and the solid line shows the flexible calibration using Loess smoothing, with the shaded area indicating the 95% confidence interval

The calibration plots, shown in Fig. 4, illustrate the association between the predicted and observed probabilities for the cardiac-origin (Panel A) and noncardiac-origin (Panel B) cohorts. The calibration curve closely followed the ideal line across the range of predicted probabilities, indicating good model calibration, for the cardiac-origin cohort. The curve slightly deviated from the ideal line at higher probabilities, but overall, the model exhibited accurate predictions. These plots are shown separately to describe model behavior within each etiologic cohort; differences in curve shapes should be interpreted descriptively in light of cohort-specific case-mix rather than as a direct between-cohort comparison.

The calibration curve initially followed the ideal line but deviated more noticeably as the predicted probabilities increased, particularly in 0.6–1.0, where the observed probabilities were lower than predicted in the noncardiac origin cohort (Panel B). This indicated a slight overestimation of the high predicted probabilities in the noncardiac-origin cohort. The gray shaded area indicates the 95% CI for both panels, demonstrating greater uncertainty at higher predicted probabilities.

## Discussion

### Summary of the study

The CRASS score in this study may be more applicable to patients with cardiac-origin OHCA (as the proportion of cardiac-origin patients in Seewald et al.’s study was as high as 76.8%) [13], such a high proportion may not be generalizable to other regions. Our strength lies in carefully conducting the external validation of the CRASS score separately for both patients with cardiac-origin and noncardiac-origin OHCA. The results revealed the higher AUROC value for CRASS was in the cardiac-origin cohort, and the score performed significantly better in predicting good neurological outcomes compared with the noncardiac-origin cohort. Furthermore, the relatively poor predictive performance of the CRASS score in non-cardiac-origin OHCA highlights its limited ability to account for the heterogeneous pathophysiology of this population. Many crucial predictors—such as indicators of infection, neurological insult, or hypoxic burden—were not part of the original CRASS variable set, which was primarily designed for cardiac-origin arrests.

### Comparison with previous external validations of the CRASS score

Our findings extended prior evidence on the external validity of the CRASS score. The only previous Asian validation, by Liu et al. (2022), reported an AUROC of 0.77 for predicting good neurological outcomes [14], comparable to our value of 0.770 in the cardiac-origin cohort. However, Liu et al. adapted several variable definitions—such as excluding the PES variable—and relied on registry data rather than independently curated medical records. In contrast, our study adhered strictly to the original CRASS variable definitions and distinguished between cardiac- and noncardiac-origin OHCA, revealing that predictive performance differs substantially by etiology. This reinforces that CRASS performs better in cardiac-origin cases, where the variable composition aligns with its development dataset, and may overestimate recovery potential in noncardiac-origin arrests.

### Clinical implications in cardiac-origin OHCAs

Our AUROC value was 0.770 (0.733–0.807) in patients with cardiac-origin OHCA, which was slightly lower than the original value of 0.88 (0.86–0.90) reported by Seewald et al. This difference may be because the proportion of cardiac-origin patients in our cohort was 50.9%, compared to 76.8% in Seewald’s study [13]. First, we utilized a rigorous process to identify the cause of OHCA, unlike registry-based studies. Additionally, a cardiac-origin proportion of > 60% is uncommon in most studies [24, 25]. Second, the population that was used to develop the CRASS score included 40.7% with a shockable rhythm, which was higher than the 25% in our cardiac-origin cohort and the typical 10%–20% seen in most studies [8, 26]. Our AUROC did not reach the 0.88 value reported in the original study may be because of the discrepancy in the shockable rhythm proportions. In summary, the closer a cohort was to purely cardiac-origin factors, the better the CRASS score’s predictive performance.

Additionally, the investigation of the performance metrics based on the optimal Youden Index revealed that a CRASS cut-off value of 0.6 provided the best statistical performance (sensitivity: 81.74 and specificity: 58.81). However, at this threshold, LR + and LR were not clinically decisive. We selected a cut-off score of 1.45, one standard deviation above the mean, which yielded an LR + of 4.23, indicating an acceptable clinical predictive value for good neurological outcomes (Supplementary Tables 3 and 4). Specifically, patients with a CRASS score that predicts a good neurological outcome are 4.2 times more likely to be discharged with a good neurological outcome (alert consciousness) than with a poor neurological outcome (vegetative state). Moreover, the specificity was 90.85 (87.74–93.38) at this threshold.

### Study limitations

This study has several limitations. First, all participating hospitals are part of a single university hospital system, which may cause a concentration of specific patient populations, such as a higher proportion of patients with cancer, despite being a multicenter retrospective study. Future studies need to consider including data from multiple hospitals across different healthcare systems to minimize potential selection bias. Second, although our results indicated that both aggressive treatment and life-sustaining treatment withdrawal would be considered depending on the population and chosen cut-off points, non-medical factors should also be discussed on a case-by-case [27]. Third, given the difference in predictive performance between cardiac-origin and noncardiac-origin OHCA cases, future research should consider recalibrating or developing modified CRASS-based models specifically for noncardiac-origin populations using large-scale datasets. Such an approach could enhance predictive accuracy and clinical applicability. Fourth, prehospital adrenaline administration may be influenced by system-level factors. Medication use is restricted by a tiered EMS certification structure, as only EMTPs can administer drugs in the field and not all ambulance crews include an EMTP. In addition, Taiwan’s high hospital density and typically short transport times—particularly in urban areas—may limit opportunities for prehospital drug delivery. Accordingly, the ‘prehospital adrenaline’ component of the CRASS score may partly reflect differences in EMS organization and transport context rather than variations in adherence to recommended care. Finally, differences in post-resuscitation management between cardiac- and noncardiac-origin OHCAs were not captured in this study. Although the CRASS score was designed as a pre-clinical model, patients with cardiac-origin OHCA—particularly those with STEMI—may have received specific interventions such as percutaneous coronary intervention (PCI) or targeted hemodynamic support that could improve survival beyond the model’s predictions. Future studies should consider incorporating treatment-related variables when validating or recalibrating prognostic scores.

## Conclusion

Among Taiwanese patients with OHCA, the CRASS score showed acceptable discrimination for predicting good neurological outcome in both cardiac-origin and non-cardiac-origin cohorts. The CRASS may be applied across etiologic subgroups for early prognostic assessment; however, interpretation should consider etiology-specific context, particularly because calibration suggested greater uncertainty and potential overestimation at higher predicted probabilities in the non-cardiac-origin cohort.

## Supplementary Information

Below is the link to the electronic supplementary material.

Supplementary Material 1

Supplementary Material 2

## Data Availability

The raw data is available on request to the corresponding author.
