# Supplementary figures and images for: External validation of the CRASS score for predicting good neurological outcome in out-of-hospital cardiac arrest: analysis from cardiac-origin and non-cardiac origin cohorts
Source: BMC Emerg Med. 2026 Jan 16;26:52. doi: 10.1186/s12873-026-01472-4 (PMC12896332; doi:10.1186/s12873-026-01472-4)

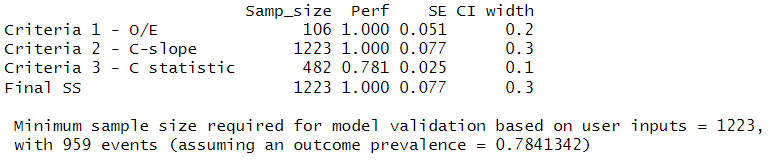

Supplement: Supplementary file 1 — Supplementary Material 1 [file 12873_2026_1472_MOESM1_ESM.png]
