# Supplementary material for: External validation of the CRASS score for predicting good neurological outcome in out-of-hospital cardiac arrest: analysis from cardiac-origin and non-cardiac origin cohorts
Source: BMC Emerg Med. 2026 Jan 16;26:52. doi: 10.1186/s12873-026-01472-4 (PMC12896332; doi:10.1186/s12873-026-01472-4)
Supplement: Supplementary file 2 — Supplementary Material 2 [file 12873_2026_1472_MOESM2_ESM.docx]

| **Supplementary Table 1**. The 22 common pre-existing diseases in Taiwan | | |
| --- | --- | --- |
| Hypertension | Hyperlipidemia | Chronic obstructive pulmonary disease (COPD) |
| Diabetes mellitus | Urinary incompetence | Non-COPD lung disease |
| Coronary artery disease | Cerebrovascular accidence | Hepatitis B and C |
| Cardiac arrythmia | Peptic ulcer disease | Liver cirrhosis |
| Congestive heart failure | Benign prostate hypertrophy | Seizure |
| Psychiatric disease | Cancers | COVID-19 |
| Arthritis | Gout |  |
| Chronic kidney disease (hemodialysis) | Acquired immunodeficiency syndrome |  |

| **Supplementary Table 2.** Comparison of the multivariable logistic regression models for factors: cardiac origin vs. non-cardiac origin cardiac arrest cohorts | | | | | | |
| --- | --- | --- | --- | --- | --- | --- |
|  | Cohort 1: cardiac origin | | | Cohort 2: non-cardiac origin | | |
| Variables | aOR | 95% CI | *p* | aOR | 95% CI | *p* |
| Age |  |  |  |  |  |  |
| ≤ 60 years | Ref. | - | - | Ref. | - | - |
| 61 – 70 years | 1.10 | 0.68 - 1.77 | 0.693 | 0.55 | 0.26 - 1.19 | 0.128 |
| 71 – 80 years | 0.64 | 0.36 - 1.13 | 0.124 | 0.34 | 0.14 - 0.87 | 0.023 |
| 81 – 90 years | 0.32 | 0.14 - 0.76 | 0.009 | 0.15 | 0.05 - 0.45 | 0.001 |
| > 91 years | 0.16 | 0.03 - 0.87 | 0.034 | 0.17 | 0.02 - 1.47 | 0.108 |
| Pre-arrest heath condition |  |  |  |  |  |  |
| with relevant disease | Ref. | - | - | Ref. | - | - |
| Without prior disease | 1.00 | 0.52 - 1.94 | 0.991 | 0.84 | 0.33 - 2.18 | 0.727 |
| With minor disease | 1.18 | 0.77 - 1.82 | 0.441 | 1.14 | 0.55 - 2.34 | 0.724 |
| Location of arrest |  |  |  |  |  |  |
| Home | Ref. | - | - | Ref. | - | - |
| Nursing home | 1.32 | 0.61 - 2.87 | 0.485 | 2.24 | 0.94 - 5.33 | 0.068 |
| Working place/sport facility | 1.69 | 1.07 - 2.67 | 0.026 | 1.24 | 0.59 - 2.63 | 0.572 |
| Public place | 1.17 | 0.59 - 2.34 | 0.648 | 0.86 | 0.10 - 7.18 | 0.890 |
| Witnessed arrest | 1.64 | 1.01 - 2.65 | 0.044 | 1.62 | 0.82 - 3.23 | 0.168 |
| Initial cardiac rhythm |  |  |  |  |  |  |
| VF/pVT | Ref. | - | - | Ref. | - | - |
| PEA | 0.34 | 0.22 - 0.53 | <0.001 | 0.68 | 0.35 - 1.30 | 0.241 |
| Asystole | 0.04 | 0.01 - 0.11 | <0.001 | 0.19 | 0.07 - 0.54 | 0.002 |
| Collapse-to-CPR interval |  |  |  |  |  |  |
| 0 – 1min | Ref. | - | - | Ref. | - | - |
| 2 – 9 min | 1.13 | 0.65 - 1.97 | 0.672 | 0.65 | 0.32 - 1.32 | 0.237 |
| ≧10mins | 0.95 | 0.48 - 1.89 | 0.883 | 0.48 | 0.16 - 1.41 | 0.181 |
| CPR duration≦5mins | 3.54 | 1.64 - 7.60 | 0.001 | 2.41 | 0.98 - 5.93 | 0.055 |
| Mechanical CPR | 0.38 | 0.22 - 0.64 | <0.001 | 0.74 | 0.36 - 1.50 | 0.402 |
| Prehospital adrenaline |  |  |  |  |  |  |
| No adrenaline | Ref. | - | - | Ref. | - | - |
| < 2mg | 1.70 | 0.57 - 5.09 | 0.345 | 1.24 | 0.11 - 13.71 | 0.858 |
| 2 – 3mg | 0.57 | 0.28 - 1.12 | 0.103 | 0.47 | 0.10 - 2.30 | 0.354 |
| 4 – 5mg | 0.19 | 0.03 - 1.09 | 0.063 | 0.98 | 0.19 - 4.94 | 0.981 |
| 6 – 7mg | 0.39 | 0.04 - 3.98 | 0.425 |  |  |  |
| ≧ 8mg | 0.08 | 0.01 - 1.25 | 0.072 |  |  |  |
| Prehospital amiodarone | 19.46 | 3.93 - 96.41 | <0.001 |  |  |  |
| SBP > 90 mmHg at admission | 2.48 | 1.16 - 5.31 | 0.019 | 2.43 | 0.69 - 8.59 | 0.169 |
| CI: confidence interval; CPR: cardiopulmonary resuscitation; CRASS: CaRdiac-Arrest-Survival-Score; aOR: adjusted odds ratio; PEA: pulseless electrical activity; SBP: systolic blood pressure; VF: ventricular fibrillation; VT: ventricular tachycardia. | | | | | | |

| **Supplementary Table 3.** Model performance in cardiac and non-cardiac origin cardiac arrest cohorts | | | | | | |
| --- | --- | --- | --- | --- | --- | --- |
|  | Cohort 1: cardiac origin | | | Cohort 2: non-cardia origin | | |
|  | Score > 0.05 | Score > 1.45* | Score > -1.35† | Score > -0.20 | Score > 1.07* | Score > -1.47† |
| N | 368 | 129 | 571 | 268 | 67 | 510 |
| Good outcome (n, %) | 188 (51.1) | 89 (68.9) | 224 (39.2) | 38 (14.2) | 18 (26.9) | 52 (10.2) |
| Sensitivity (95% CI) | 81.74 (76.13, 86.51) | 38.70 (32.37, 45.32) | 97.39 (94.41, 99.04) | 71.70 (57.65, 83.21) | 33.96 (21.52, 48.27) | 98.11 (89.93, 99.95) |
| Specificity (95% CI) | 58.81 (54.03, 63.47) | 90.85 (87.74, 93.38) | 20.59 (16.90, 24.69) | 61.08 (57.02, 65.03) | 91.71 (89.19, 93.80) | 22.50 (19.20, 26.09) |
| PPV (95% CI) | 51.09 (45.85, 56.30) | 68.99 (60.25, 76.84) | 39.23 (35.20, 43.37) | 14.18 (10.23, 18.94) | 26.87 (16.76, 39.10) | 10.20 (7.71, 13.16) |
| NPV (95% CI) | 85.95 (81.49, 89.69) | 73.79 (69.86, 77.46) | 93.75 (86.89, 97.67) | 96.01 (93.51, 97.75) | 93.93 (91.66, 95.74) | 99.25 (95.91, 99.98) |
| LR^+^  (95% CI) | 1.98 (1.75, 2.25) | 4.23 (3.02, 5.92) | 1.23 (1.16, 1.29) | 1.84 (1.51, 2.24) | 4.10 (2.58, 6.50) | 1.27 (1.20, 1.34) |
| LR^-^  (95% CI) | 0.31 (0.23, 0.41) | 0.67 (0.61, 0.75) | 0.13 (0.06, 0.29) | 0.46 (0.30, 0.71) | 0.72 (0.59, 0.87) | 0.08 (0.01, 0.59) |
| CI: confidence interval; LR^+^: positive likelihood ratio; LR^-^: negative likelihood ratio; NPV: negative predictive value; PPV: positive predictive value. *: Best cut-off value plus one standard deviation  †: Best cut-off value minus one standard deviation  The standard deviation is 1.4 in the cardiac origin cohort, and 1.27 in the non-cardiac origin cohort. | | | | | | |

| **Supplementary Table 4.** The detailed values for the performance metrics | | | | | |
| --- | --- | --- | --- | --- | --- |
|  | Cut-off * | Sensitivity | Specificity | PPV | NPV |
| Cardiac-origin | -1.7 | 0.99 | 0.16 | 0.38 | 0.96 |
|  | -1 | 0.94 | 0.29 | 0.41 | 0.91 |
|  | -0.6 | 0.90 | 0.40 | 0.44 | 0.88 |
|  | -0.1 | 0.82 | 0.53 | 0.48 | 0.85 |
|  | 0.05 | 0.81 | 0.59 | 0.51 | 0.86 |
|  | 0.2 | 0.76 | 0.63 | 0.52 | 0.83 |
|  | 0.5 | 0.64 | 0.73 | 0.56 | 0.79 |
|  | 1 | 0.50 | 0.84 | 0.63 | 0.76 |
|  | 1.4 | 0.40 | 0.90 | 0.68 | 0.74 |
|  | 1.9 | 0.24 | 0.97 | 0.80 | 0.71 |
| Non-cardiac origin | -2.10 | 1.00 | 0.11 | 0.09 | 1.00 |
|  | -1.50 | 1.00 | 0.21 | 0.10 | 1.00 |
|  | -1.00 | 0.91 | 0.32 | 0.11 | 0.97 |
|  | -0.70 | 0.85 | 0.45 | 0.12 | 0.97 |
|  | -0.50 | 0.77 | 0.50 | 0.12 | 0.96 |
|  | -0.20 | 0.72 | 0.61 | 0.14 | 0.96 |
|  | 0.10 | 0.58 | 0.70 | 0.15 | 0.95 |
|  | 0.60 | 0.49 | 0.82 | 0.19 | 0.95 |
|  | 1.10 | 0.32 | 0.92 | 0.27 | 0.94 |
| *The determination of the cut-off values was based on the 10th, 20th, 30th, ..., up to the 90th percentile, plus the optimal cut-off, resulting in a total of ten points. However, in the non-cardiac origin cohort, the optimal cut-off value fell exactly at the 60th percentile, leaving only nine points.  PPV: positive predictive value; NPV: negative predictive value. | | | | | |
